# Molecular Determination of Tumor Necrosis Factor-alpha, Interleukin-8, Interleukin-10, and C-X-C Chemokine Receptor-2 Genetic Variations and their Association with Disease Susceptibility and Mortality in COVID-19 Patients

**DOI:** 10.2174/0113892029272497240103052359

**Published:** 2024-01-17

**Authors:** Badr A. Alsayed, Rashid Mir, Mohammad M. Mir, Tarig M.S. Alnour, Shereen Fawzy, Mesaik M. Ahmed, Dnyanesh Amle

**Affiliations:** 1 Department of Internal Medicine, Faculty of Medicine, University of Tabuk, Tabuk, 71491, Saudi Arabia;; 2 Department of Medical Lab technology Prince Fahad Bin Sultan Chair for Biomedical Research, Faculty of Applied Medical Sciences, University of Tabuk, Tabuk, 71491, Saudi Arabia;; 3 Department of Basic Medical Sciences (Biochemistry), College of Medicine, University of Bisha, Bisha, 61922, Saudi Arabia;; 4 Department of Medical Microbiology, Faculty of Medicine, University of Tabuk, Tabuk, 71491, Saudi Arabia;; 5 Department of Biochemistry, All India Institute of Medical Sciences, Nagpur, 441108, India

**Keywords:** Coronavirus disease-2019 (COVID-19), interleukin 8 (IL-8), tumor necrosis factor alpha (TNF-α), interleukin 10 (IL-10), CXC chemokine receptor-2 (CXCR-2), genetic polymorphisms

## Abstract

**Background:**

Altered cytokine levels have been associated with poor outcomes among COVID-19 patients. TNF-α, IL-8 and IL-10 are key cytokines in COVID-19 pathogenesis, and CXCR-2 is a major chemokine receptor involved in inflammatory response. Polymorphisms in the genes of these proteins are proposed to influence disease outcomes. In this study, we aimed to find out the association of genetic polymorphisms in TNF-α, IL-8, IL-10 and CXCR-2 genes with susceptibility to and mortality of COVID-19.

**Methods:**

The present case-control study was conducted on 230 subjects, among whom 115 were clinically diagnosed and RT-PCR-confirmed COVID-19 patients and 115 healthy control subjects. The polymorphisms in TNFα -308 G>A (rs1800629), IL-8 -251T>A (rs4073), CXCR2 +785 C>T (rs2230054) genes were detected by ARMS -PCR assay whereas for IL-10 (-1082 G>A), rs1800896 G>A allele-specific PCR assay was used and their association with COVID-19 susceptibility and mortality was estimated by multivariate analysis. The results were analyzed for risk of infection and mortality through different inheritance models.

**Results:**

Frequencies of TNF-α rs1800629 GA, AA, IL-8 rs4073 TA, AA, IL-10 (-1082 G>A), rs1800896 GA and GG, and CXCR2 rs2230054 CT genotypes were significantly higher in COVID-19 patients compared to the control group (*p* < 0.05). Furthermore, COVID-19 patients had a higher frequency of the polymorphic A allele of TNF-α, the A allele of IL-8, the G allele of IL-10, and the T allele of CXCR2. The risk of susceptibility to COVID-19 was significantly associated with TNF-α rs1800629 GA, GA+AA genotypes and the A allele, IL-8 rs4073 TA, AA genotypes and A allele, IL-10 rs1800872 GA and CC genotypes and C allele, and CXCR2 rs2230054 CT and CT+CC genotypes. TNF-α-GA and AA genotypes and A allele, IL-8 TA and AA genotypes and A allele and CXCR-2 CC and CT genotypes have significant associations with mortality risk in COVID-19 patients, while GA and GG genotypes of the IL-10 are shown to confer significant protection against mortality from COVID-19.

**Conclusion:**

The findings of this study provide important insights into the COVID-19 disease and susceptibility risk. The polymorphisms in TNFα -308 G>A (rs1800629), IL-8 -251T>A (rs4073), IL-10 (-1082 G>A), rs1800896 and CXCR2 +785 C>T (rs2230054) are associated with the risk of susceptibility to COVID-19 and with mortality in COVID-19 patients. Further studies with larger sample sizes are necessary to confirm our findings.

## 1 INTRODUCTION

Coronavirus disease-2019 (COVID-19) is an infectious disease caused by the Severe acute respiratory syndrome Coronavirus -2 (SARS-COV-2). SARS-CoV-2 is estimated to have infected 762.2 million people around the globe and has claimed approximately 6.82 million lives worldwide [1]. With progress of the pandemic through multiple epidemiological phases, the virus has evolved into variants *viz*. Alpha, Beta, Delta, Omicron *etc*, each with different epidemiological properties and virulence but a relatively common mechanism to cause complications [2, 3]. It has been observed that altered levels of various cytokines *viz*. interleukin-6 (IL-6), IL-10, tumor necrosis factor (TNF)-α, IL-1β, IL-4, IL-8 and IL-17 are associated with increased severity as well as mortality in COVID-19 patients [4]. Also, increased expression of CXC chemokine receptor 2 (CXCR2) has been found to be associated with increased severity and mortality in COVID-19 patients [5]. Most of the cytokine genes are polymorphic with single-nucleotide polymorphisms (SNPs). In healthy individuals, there are stable and reproducible differences in cytokine production, and these differences are linked with genetic variations in the encoding genes [6].

### 1.1 Tumor Necrosis Factor Alpha (TNF-α) and COVID-19

TNF-α has a fundamental role in almost all acute inflammatory responses as an amplifier and even as a coordinator of inflammation [7]. TNF-α is detected in the blood and tissues of patients with COVID-19, associated with bronchial hyperresponsiveness and is linked with the reduction of airway caliber and enhanced neutrophilia in the epithelium of the respiratory tract [6]. The promoter zone of the TNF-α gene is a polymorphic region, and it has been found to be associated with various inflammatory diseases and increased susceptibility to some infections [7, 8]. The rs1800629 G>A is the most common single nucleotide polymorphism (SNP) in this region, and it has been shown to cause increased transcription and is linked to heightened immune response [9-14]. A long-lasting cytokine signature with high levels of interleukin (IL)-1, IL-6, and tumour necrosis factor (TNF) may be responsible for many of the clinical signs of Post-acute sequelae of COVID-19 (PASC) and may originate in the macrophage compartment, according to one study [15].

### 1.2 Interleukin-8 (IL-8) and COVID-19

Interleukin-8 (IL-8) is another pro-inflammatory cytokine that has a role in neutrophil activation and has been identified to play a critical role in the pathogenesis and progression of COVID-19. IL-8 rs4073 T>A polymorphism is associated with increased transcription activity of the IL-8 gene, thus associated with higher levels of IL-8 [16]. Increased levels of IL-8 have been associated with increased severity and mortality of COVID-19 [17]. Cellular activity of IL8 is mediated by the receptor CXCR2. The CXCR2 is expressed in immune cells, including neutrophils, mast cells, monocytes and macrophages [17, 18]. It is also found in endothelial and epithelial cells and numerous types of tumour cells. Because of IL-8's critical involvement in lung disease aetiology and inflammation, it may be conceivable to use IL-8 as a new therapeutic target to effectively control the hyperinflammatory response in ARDS [19]. Dynamic fluctuations in serum IL-6, IL-8, and IL-10 levels were linked to patient survival in the intensive care unit and may be used as a biomarker to predict treatment options for COVID-19 patients [20]. An important pathogenic cytokine linking systemic hyper-inflammation to the clinical results of COVID-19 is plasma IL-8, according to a recent study [21].

### 1.3 CXC Chemokine Receptor-2 (CXCR-2) and COVID-19

CXCR2 +785 C>T (rs2230054) polymorphism located in exon 11 is proposed to result in a silent codon change. Despite the silent codon changes, the polymorphism has been associated with an increased risk of multiple diseases through the alteration of inflammatory modulators [7]. Taking into consideration that CXCR2 binds several chemokines, including IL-8, that trigger its function, TNF-α and IL-8 are important cytokines involved in the inflammation of the airways. In response to inflammatory stimuli, macrophages release the TNF-α, which in turn triggers the release of IL-8 and controls its transcription. Thus, the interdependence and molecular interaction of these three molecules highlight their candidacy for studying different inflammatory processes [22, 23]. Hence, evaluation of these molecules and their genetic variability in the population may help to determine the risk of morbidity and mortality in COVID-19 patients.

### 1.4 Interleukin-10 (IL-10) and COVID-19

Interleukin-10 (IL-10) is known for its action inhibition of differentiation and suppression of TH1 cells [24]. IL-10 (-1082 G>A), rs1800896 polymorphism has been associated with lower levels of IL-10 [25]. Three polymorphisms, IL10 rs1800871 (− 819 T/C), rs1800872 (− 592 C/A), and rs1800896 (-1082 G/A), in the promoter region of the IL10 gene have been studied to date. Their haplotypes in different populations are related to the low or high expression of the IL10 gene [26]. Polymorphisms in the promoter region contribute genetically to interindividual variations in IL10 production. The IL10 rs1800896 (-1082 G/A) polymorphism has been found to be associated with greater IL10 serum levels and an increased risk of developing severe pneumonia [27]. Populations in Japan, China, Tunisia, and Mexico typically have the AA genotype, according to the IL-10 gene polymorphisms in various populations at the rs1800896 locus [28]; however, the AG genotype is widely found in populations of Iran, India, the Netherlands, Finland, Germany, Spain, Czechia, Norway, Poland, the UK, and Brazil [29]. Only among the Italian population, the rs1800896 GG genotype had the highest frequency [30]. The aim of this study is to evaluate the association of TNFα -308 G>A (rs1800629), IL-8 -251T>A (rs4073), IL-10 (-1082 G>A), rs1800896, and CXCR2 +785 C>T (rs2230054) gene polymorphisms with the susceptibility and mortality of COVID-19, as limited studies have been reported from this part of the globe concerning this issue.

## 2 MATERIALS AND METHODS

### 2.1 Study Population

The present case-control study was composed of 230 subjects divided into two groups patient group and healthy control group. Patient group was composed of 115 COVID-19 patients, and the control group was composed of 115 healthy controls collected from two cities in Saudi Arabia: Tabuk and Bisha (Asir), as highlighted in Fig. (**1**). The study was conducted from April 2020 to December 2022.

#### 2.1.1 Patients’ Group

Patient group: It included 115 COVID-19 patients admitted to two tertiary care hospitals in Saudi Arabia. Over the study period, a total of 70 and 45 consecutive admissions from King Fahad Hospital, Tabuk City, and King Abdullah Hospital, Bisha City, respectively, were included. A positive result on nasal and oropharyngeal swab samples tested with the SARS-CoV-2 RT-qPCR detection kit was considered the laboratory confirmation of COVID-19.

#### 2.1.2 Control Group

This included 115 control group subjects selected among subjects with a high rate of exposure to the SARS-CoV-2 and included subjects having a family history of COVID-19 and/or healthcare workers with high exposure to COVID-19 patients but tested multiple times and showed a negative result for SARS-CoV-2 RNA by RT-PCR test in the routine hospital lab.

### 2.2 Ethical Approvals

In accordance with regional regulations that broadly complied with the principles of the Helsinki Declaration, the ethical approvals were obtained from three local institutional ethics committees at the University of Tabuk (Decision no. KAEK2020/4/4), the University of Bisha (Ref. No. UBCOM/H-06-BH-087(05/25), and the King Khalid University, Abha (Ref. no. H-06-B-091). All the subjects provided written informed consent before their participation in the study.

### 2.3 Sample Collection

A peripheral blood sample of around 2 mL was collected from the 230 subjects in an EDTA tube for the purpose of genotyping and kept in storage right away at -20°C till analysis. Using structured questionnaire, all included subjects were interviewed with regard to epidemiological and demographic information, history of coronary artery disease (CAD), type 2 diabetes mellitus (T2DM), chronic kidney disease (CKD) and addictions, particularly smoking. Also, a family history of any other important illnesses was documented.

### 2.4 Genomic DNA Extraction

Using DNeasy Blood K (Qiagen), genomic DNA was extracted in accordance with the manufacturer's instructions. The isolated DNA was dissolved in nuclease-free water and kept at 4°C until needed. DNA quality and integrity were examined using NanoDrop^TM^ (Thermo Scientific, USA). By measuring optical density (OD) at 260 nm (OD260) and 280 nm, all DNA samples from COVID-19 and controls were checked for purity (OD280). The range of 1.83 to 1.99 in the 260/280 ratios indicated high-quality DNA.

### 2.5 Genotyping of TNF-α, IL-8, IL-10 and CXCR2

#### 2.5.1 Preparation of PCR Cocktail

The PCR was performed in a reaction mixture total volume of 12 µL containing template DNA (50 ng), Fo-12.5 µL, Ro—12.5 µL, FI-12.5 µL, RI—12.5 µL of 25 pmol of each primer (Table **1**) and 6 µL from GoTaq^®^ Green Master Mix (M7122) (DreamTaq Green, Thermo Fisher Scientific, Inc.) The final volume was adjusted to 12 µL by adding nuclease-free double distilled water (ddH_2_O). Two µL of DNA from each subject was added in the end.

#### 2.5.2 Thermocycling Conditions

##### 2.5.2.1 For TNFα -308 G>A, IL-8 T>A,CXCR2-C>T

The thermocycling conditions used were: 95°C for 8 min followed by 30 cycles of 95°C for 32 s, annealing temperature; (TNFα -308 G>A (rs1800629) (62°C), IL-8T>A (rs4073) (58°C), and CXCR2 +785 C>T (rs2230054) (63°C)) for 35s, extension 72°C for 40 s followed by the final extension at 72°C for 08 min and storage at 4°C.

##### 2.5.2.2 For IL-10 (-1082 G>A), rs1800896

The cycling conditions for IL-10 (-1082 G>A), rs1800896 of PCR were as follows: 94°C for 3 min (1 cycle), followed by 96°C for 25 s, 70°C for 45 s, and 72°C for 20 s (5 cycles); followed by 96°C for 25 s, 65°C for 50 s, and 72°C for 45 s (11 cycles); and finally 96°C for 25 s, 55°C for 60 s, and 72°C for 2 min (15 cycles).

### 2.6 Gel Electrophoresis of SNP Amplifications

PCR products obtained were separated on 2.5% agarose gel using 2 µL of sybre safe stain (Thermo Scientific, Waltham, MA, USA), and the bands thus obtained were visualized on a UV-trans illuminator, Bio-Rad (Hercules, CA, USA). The size of PCR products was determined relative to the migration of a 100 bp step ladder (Fermentas).

#### 2.6.1 TNFα -308 G>A (rs1800629)

To flank the exon/intron of the TNF- gene, the primers FO and RO were created, which produced a 323 bp band. This band functioned as a check to determine whether the DNA was intact. Moreover, a 224 bp band corresponding to the G allele was produced when the primers FO and RI were employed. As an alternative, the A allele's 154 bp band was generated by primers FI and RO.

#### 2.6.2 IL-8 -251 T>A (rs4073)

A 349 bp band was produced as a control for assessing the DNA's quality when the exterior portion of the IL-8 gene was amplified using the external primers FO and RO. The 228 bp band produced by primers FO and RI corresponds to the T allele, while the 169 bp band produced by primers FI and RO corresponds to the A allele.

#### 2.6.3 CXCR2 +785 C>T (rs2230054)

The CXCR2 gene's external region was amplified using the external primers FO and RO, yielding a 451 bp band that served as a control for assessing the DNA's integrity. A 281 bp band belonging to the T allele was created by primers FO and RI, while a 226 bp band corresponding to the C allele was produced by primers FI and RO.

#### 2.6.4 IL-10 (-1082 G>A), rs1800896

After amplification, the AS-PCR products of the control primer resulted in an amplicon of 429 bp, and the − 1082 primers resulted in an amplicon of 258 bp for G allele and 258 bp for A allele. By 2.5% agarose gel, the size of PCR products was determined relative to the migration of a 100 bp step ladder (Simply).

### 2.7 Statistical Analysis

Chi-square (χ^2^) goodness-of-fit test was used to calculate deviations from Hardy-Weinberg disequilibrium (HWD). Student's two-sample t-test and one-way analysis of variance (ANOVA) with Tukey's post hoc test were used to compare group differences for continuous variables and χ^2^ for categorical variables. χ^2^ test was used to evaluate differences in the TNF-α rs1800629 G>A, IL-8 rs4073 T>A, IL-10 rs1800896 G>A and CXCR2 rs2230054 C>T allele and genotype frequencies between groups. The associations between TNF-α rs1800629 G>A, IL-8 rs4073 T>A, IL-10 (-1082 G>A), rs1800896, CXCR2 rs2230054 C>T genotypes and odd ratios (ORs) and risk ratios (RRs) with 95% confidence intervals (CIs), were used to assess the susceptibility to COVID-19. The Hardy-Weinberg equilibrium (HWE) test was used to compare allele frequencies in controls. With SPSS 16.0, all statistical evaluations were carried out (SPSS, Inc.). The threshold for statistical significance was set at *P* < 0.05.

## 3 RESULTS

### 3.1 Demographic Features

The listed demographic features of the 115 consecutive COVID-19 patients, associated comorbid factors, biochemical markers and therapy are summarized in Table **2**. Out of the 115 COVID-19 patients included in the study, 51 (44.3%) succumbed. Male patients were at higher risk of failure to recover than female patients (OR 0.4435 (0.20-0.96). Type 2 diabetes mellitus posed the highest risk (OR 22.75 (8.5078- 60.834) followed by hypertension (14.9833 (5.4507-41.1872) and chronic kidney disease (6.6429 (1.3662-32.2985). However, no significant difference was detected between the survivor and non survivor groups regarding the age (Table **3**).

### 3.2 Hardy–Weinberg Equilibrium for Genotype Distributions and Allele Frequencies

The genotype distributions and allele frequencies of the SNPs for the four genes showed little or no variation. The control group had TNF-α rs1800629 G>A, IL-8 rs4073 T>A, IL-10 rs1800896 G>A, and CXCR2 rs2230054 C>T genotypes. There was no deviation from the Hardy–Weinberg equilibrium for the control group in the genotype distributions. Allele frequencies of the SNPs located in the genes for four gene TNF-α, IL-8, IL-10 and CXCR2 were as follows: for TNF-α rs1800629, G>A gene polymorphism HWE was (χ^2^ = 0.55 *p <* 0.45), for IL-8 rs4073 T>A HWE, it was (χ^2^ = 3.62 *p <* 0.05), for IL-10 rs1800896 G>A HWE, it was (χ^2^ = 0.70 *p <* 0.40) and for CXCR2 rs2230054 C>T HWE, it was (χ^2^ = 3.51 *p* < 0.06). As a result, we randomly selected 10% of the samples from the normal control group to review the genotyping results, demonstrating that the accuracy rate was greater than 99%. As a result, the genotyping findings from 10% of the samples randomly selected from the healthy control group were evaluated. These results showed an accuracy of more than 99%. *P*-values > 0.05 were considered significant.

### 3.3 Genotype Association of TNF-α rs1800629 G>A Gene Polymorphism in COVID-19 Patients

A significant statistical difference was found between COVID-19 patients and the control group regarding the frequency of TNF-rs1800629 G>A genotypes (*P* = 0.025), where the GA genotype was significantly higher in the patient group (30.43%) than the control group (17.39%). Also, it was found that COVID-19 patients were more likely to carry the A allele than healthy controls (0.20 *vs*. 0.10) (Table **4**). TNF-α rs1800629 GA genotype showed a significant association with susceptibility to COVID-19 patients (OR= 2.17, 95% CI = 1.158 to 4.066, RR=1.42, and *P* = 0.01) in the codominant model. In the dominant inheritance model, TNF-α (GA+AA) genotype showed a significant association with susceptibility to Covid-19 (OR=2.25, 95% CI= 1.234-4.119, RR=1.44 and *P* = 0.005). However, in the recessive inheritance model, TNF-α (GG+GA) and AA genotypes failed to reach statistical significance. In allelic comparison, the TNF-α-A allele was found to be significantly associated with susceptibility to COVID-19 (OR of 2.08, 95% CI=1.22 to 560, RR 1.37, and *P* = 0.006). In the over-dominant inheritance model, a statistically significant association was noted between TNF-α GA genotype and susceptibility to COVID-19 (OR=2.07, 95% CI= 1.112 to 3.81, RR=1.39 and *P* = 0.021) (Table **5**).

### 3.4 Genotype Association of IL-8 rs4073 T>A Gene Variants in COVID-19 Patients

A significant statistical difference was found between COVID-19 patients and the control group regarding the frequency of IL-8 rs4073 T>A genotypes (*p* = 0.044). The frequency of TA (50.45%) and AA (27.92%) genotypes was higher compared to matched controls, who were more likely to have frequencies of TT (38.26%), TA (40%), and AA (21.73%). Also, it was found that COVID-19 patients were more likely to carry the A allele than healthy controls (0.53 *vs*. 0.42) (Table **4**). In the codominant model, IL-8 rs4073 TA genotype showed a significant association with susceptibility to Covid-19 (OR= 2.17, 95% CI: 1.158 - 4.087, RR=1.51, 95% CI: 1.0649 -2.1537 and *P* = 0.011). Similarly, IL-8 rs4073 AA genotype was found to be significantly associated with susceptibility to COVID-19 (OR=2.09, 95% CI = 1.0255 to 4.2939, RR=1.49 and *P* = 0.031). In the dominant inheritance model, a significant association was observed between IL-8 rs4073 IL-8 (TA+AA) genotype and susceptibility to Covid-19 (OR= 2.14, 95% CI= 1.2 to 3.83, RR= 1.505, 95% CI= 1.0761 – 2.1065, and *P* = 0.006.). In the recessive inheritance model, IL-8 -(TT+TA) and IL-8 AA genotypes did not show a significant association with susceptibility to Covid-19 (OR=1.33, 95% CI: 0.725 - 2.44, RR= 1.14, 95% CI= 0.8654 to 1.5347, *p* value=0.21). In allelic comparison, the IL-8-A allele showed a significant association with susceptibility to COVID-19 (OR= 1.54, 95% CI=1.0627 to 2.2500, RR= 1.24, and *P* = 0.014). In the over-dominant Inheritance model, no statistically significant association was noted between IL-8- (TA) genotype and susceptibility to Covid-19 (OR=1.5564, *P* = 0.067) (Table **6**).

### 3.5 Genotype Association of CXCR2 rs2230054 C>T Gene Variants in COVID-19 Patients

A significant statistical difference was found between COVID-19 patients and the control group regarding the frequency of CXCR2 rs2230054 C>T genotypes (*P* = 0.038). In COVID-19 patients, the frequency in patients was TT (26.08%), CT (66.95%), and CC (6.95%) compared to the controls, who were more likely to have frequencies of TT (41.73%), CT (51.30%), and CC (6.95%), respectively. Also, it was found that COVID-19 patients were more likely to carry the C allele than healthy controls (0.41 *vs*. 0.33) (Table **4**). In the codominant model, a statistically significant association was noted between the CXCR2 rs2230054 CT genotype and susceptibility to COVID-19 (OR 2.0, 95% CI = 1.184 to 3.681, RR = 1.41 and *p* = 0.011). However, CXCR2 rs2230054 CC genotype failed to show association with susceptibility to COVID-19 (OR=1.60, 95% CI = 0.5413 to 4.71, RR=1.23 and *p* = 0.394). In the dominant inheritance model, the CXCR2 - (CT+CC) genotype was found to be significantly associated with susceptibility to COVID-19 (OR = 2.09, 95% CI= 1.1626 to 3.5441, RR= 1.45 and *p* = 0.012). In the recessive inheritance model, the CXCR2-CC genotype failed to show a significant association with susceptibility to COVID-19 (OR =1.00, 95% CI=0.365 to 2.76, RR = 1.10, and *P* = 1.0). Also, in allelic comparison, CXCR2- neither T or C allele was found to be significantly associated with susceptibility to COVID-19 (OR = 1.13, RR=1.06 and *p* = 0.810). In the overdominant Inheritance model, a statistically significant association was noted between CXCR2 CT genotype and susceptibility to Covid-19 (OR=1.92, *p* = 0.016) (Table **7**).

### 3.6 Genotype Association of IL-10 (-1082 G>A), rs1800896 Gene Variants in COVID-19 Patients

A significant statistical difference was found between Covid-19 patients and the control group regarding the frequency of IL-10 (-1082 G>A), rs1800896 genotypes (*P* = 0.004). In Covid-19 patients, the frequency was AA (30.03%), GA (45.94%), and GG (18%) compared to matched controls who were more likely to have frequencies of AA (56.75%), GA (35.13%), and GG (8.10%), respectively. Also, it was found that COVID-19 patients were more likely to carry the G allele than healthy controls (0.41 *vs*. 0.25) (Table **4**). In the codominant model, IL-10 (-1082 G>A), rs1800896 GA genotype was found to be significantly associated with susceptibility to Covid-19 (OR= 2.05, 95% CI = 1.1587 - 3.6609, RR=1.45 and *p* = 0.009) and similarly IL-10-GG genotype (OR= 3.5 95% CI=1.4505 - 8.445, RR=1.77 and *p* = 0.003). In the dominant inheritance model, IL-10 - (GA+GG) genotype was significantly associated with susceptibility to Covid-19 (OR=2.32, 95% CI=1.358-3.996, RR=1.536 and *p* = 0.001). In the recessive inheritance model, a statistically significant association was observed between the IL-10 GG genotype and susceptibility to COVID-19 (OR=2.49, 95% CI=1.0797 - 5.746, RR 1.46, and *p* = 0.022). In the allelic comparison, the IL-10-G allele was found to be significantly associated with susceptibility to Covid-19 (OR= 2.01, 95% CI=1.34 – 3.00, RR=1.38, and *p* = < 0.001). In the over-dominant inheritance model, no statistically significant association was noted between the IL-10-(GA) genotype and susceptibility to Covid-19 (OR=1.5692, *p* = 0.066) (Table **8**).

### 3.7 Association between COVID-19 Patients with Survivors and Non-survivors

General characteristics of COVID-19 survivors and non-survivors are listed in Table **9**, subjects. The cut-off levels of age for the prediction of COVID-19 outcome were selected as ≥40. At the time of analysis, 96(83.47%) patients were aged greater than 40 years, and 19(16.52%) were less than 40 years. About 54 (56.25%) were survivors and 42 (43.75%) were non survivors. Out of 19, 10(15.62%) were survivors, and 9(17.30%) were non-survivors; however, the frequency of females in the survivor group was found to be significantly higher compared to non-survivors (*p* = 0.02). The comorbidities were found to pose a significant risk of death in COVID-19 subjects. Type 2 diabetes mellitus posed the highest risk (OR 22.75 (8.5078- 60.834)) followed by hypertension (14.9833 (5.4507-41.1872)) and chronic kidney disease (6.6429 (1.3662-32.2985). Low oxygen saturation, longer duration of hospital stay, and raised levels of biochemical markers, such as ALT and AST, were amongst other factors increasing the risk of death.

### 3.8 Haplotype Analysis TNFα, IL-8, IL-10 CXCR2 Genotypes

In the present study, the haplotype analysis was performed by using SNPstats software (http:// bioinfo.iconcologia.net/snpstats/start.htm). For SNPs SNP1, SNP2 and SNP3, 8 major haplotypes *viz*., TTG, ACG, ATG, TTA, ATA, ACA, TCG and TCA were observed (Table **10**). The test results showed that 3 haplotypes *viz*., ACG, ATA and ACA, out of 8 considered, demonstrated statistically significant association with disease. It is believed that haplotype analysis can provide more information than single-locus analysis. In the analysis of possible 8 haplotypes from different combinations of the three studied variable SNPs, 2 haplotypes *viz*., ACG and ACA were found to be linked to significant potential protective effect with disease (OR=0.03, *p* = < 0.0001; and OR=0.22, *p* = 0.02 respectively), while ATA was found to be linked to significant risk effect with disease (OR=5.38, *p* = < 0.028).

## 4 DISCUSSION

It is well established that co-morbid conditions, such as CKDs, diabetes, hypertension and CAD, increase the risk of severity of COVID-19 and lead to increased mortality among patients [31-34]. In the present study, the risk of mortality in COVID-19 patients can be partially explained by these known risk factors, including male gender and the presence of comorbidities, such as T2D, hypertension, CAD and CKD where these factors remained highly associated with the non-survivor group. However, there was no significant difference in the succumbed patients’ group regarding age, where death has also been observed in patients <40 years, suggesting that other risk factors, such as genetic variants, may increase the risk of severity of this disease. It is known that host genetic polymorphisms play a key role in the susceptibility or resistance to different viral infections [11, 12] as well as disease severity and outcome. Several studies highlighted the association between polymorphisms in cytokines/chemokines/chemokine receptors genes and Covid-19 development and/or severity [6, 9]. In the current study, we investigated the association of TNFα -308 G>A (rs1800629), IL-8 -251T>A (rs4073), IL-10 (-1082 G>A), rs1800896, and CXCR2 +785 C>T (rs2230054) gene polymorphisms with the susceptibility to and mortality of Covid-19.

### 4.1 Comparative Analysis of TNF-α-308 G > A (rs1800629) Gene Polymorphism

TNFα gene variant, rs1800629 G>A, can be present in three distinct genetic phenotypes: the homozygous form G/G (wild type) as well as the mutated version forms G/A and A/A. In comparison to the GG variant, our investigations found that the TNF-SNP's GA and AA variants are significantly more frequent in COVID-19 patients. TNF-α GA and (GA+AA) genotypes significantly increased the risk of infection by 1.42 and 1.44-fold, respectively. In addition, the A allele was associated with a significantly increased risk of infection with OR 2.08(1.22-3.5603). The GA and AA genotypes, as well as the A allele of the TNF-α rs1800629 G>A polymorphism, have been demonstrated to increase the risk of mortality from COVID-19. Our findings are consistent with those of Saleh *et al.* [14], who noted that the mutant AA genotype is predominately more common in severe COVID-19 patients. They further noted that high blood TNF- levels may be the result of the association between the A allele and more severe illness [14, 35]. Several recent studies made comparable findings as well [9, 13, 36]. Similar findings from earlier analyses of this polymorphism were obtained with other inflammatory diseases [11]. This could be clarified by the study of Silva *et al.* [37], who noted that the wild-type genetic profile G/G occurs most frequently in a defined sample of a certain group of people, typically linked to people who produce little of TNF-α. The mutated version, G/A, nevertheless, is associated with medium production, whereas the less common kind, A/A, is linked with max-level TNF-α-productive people [10]. According to allelic A *vs*. G, GA *versus* GG, and GA + AA *versus* GG inheritance models, TNF-rs1800629 is linked to an increased risk of sepsis, a systemic inflammatory response to infection [38], and also TNF-α-308 G > A (rs1800629) polymorphism may play an important role in the development of metabolic syndrome and A allele is a strong predictor in Egyptians.

### 4.2 Comparative Analysis of IL-8 rs4073-251T/A Gene Polymorphism

IL-8 has a role in inflammation, recruitment of immune cells, neutrophil activation, and degranulation [17, 18]. In SARS-CoV-2 infection, it was reported that the prothrombotic, degranulated neutrophil phenotype in severe COVID-19 is associated with increased IL-8 release and expression on neutrophils recruited to the pulmonary tissue, which in turn activates IL-8 production from peripheral neutrophils as sustained loops [6]. IL-8 has been demonstrated to be significantly higher in non-survivors compared to survivors of COVID-19, and the dynamic change of serum IL-8 levels has been correlated with the severity of the disease [17]. IL8 serum levels were associated with a reduced occurrence of MI among women, whereas IL8 was associated with a slightly increased risk among men, possibly through different mechanisms. In the region proximal to the promoter of the IL-8 gene, SNP −215 T > A rs4073 lies, which can regulate the production of IL-8 and is related to variations in gene expression and IL-8 levels [16]. In the current study, the TA and AA variant of IL-8 rs4073 T>A, as well as the A allele, showed a significantly high risk of infection and mortality compared to the TT variant and T allele respectively. To the best of our knowledge, this study is the only one that addressed the association of the IL-8 rs4073 T>A polymorphism with COVID-19 patients. However, previous studies have demonstrated a link between this gene polymorphism and increased susceptibility to infection and an inflammatory response [17, 18]. Studies have demonstrated that IL-8 rs4073-251T/A boosted IL-8 production under lipopolysaccharide stimulation and, thus, exerted an influence on various pathological conditions to a certain degree [39, 40]. It has been observed that the −251 A allele of the rs4073 polymorphism can enhance IL-8 expression [41] and levels of IL-8, with the A allele being related to higher levels of IL-8 [42, 43]. In a meta-analysis by Li *et al.* [41], they found that subjects bearing the A allele of the IL-8 rs4073 polymorphism might be more sensitive to acute pancreatitis [41]. The allele A of the IL-8 -251 T/A may also increase the risk of developing recurrent attacks after first-time acute pyelonephritis [44]. The same polymorphism is also found to be associated with the risk and mortality from sepsis [43], while the presence of IL-8 rs4073 T allele was associated with protection against TB [45]. However, Yao *et al.* [39] who investigated the association of SNPs of the IL-8 gene rs4073 (-251T>A) and its receptor CXCR2 rs2230054 (+811T>C) with SIRS in patients exposed to wasp sting injury, observed significantly higher frequencies for the IL-8 - 251T allele (AT+TT) in the SIRS group when compared with the control group. They suggested that the IL-8 - 251T allele (AT+TT) could be a risk factor among SIRS patients with wasp sting injury.

### 4.3 Comparative Analysis of CXCR2 rs2230054 C>T Gene Polymorphism

CXCR2 is an IL-8 receptor through which IL-8 exerts its effects and is a key stimulator of immune cell migration [7]. The CXCR2 rs2230054 C>T polymorphism is an SNP identified at the CXCR2 gene, leading to a 3’ UTR variant [22]. Our investigations revealed that the frequency of the CXCR2 rs2230054 C>T CT genotype is higher in COVID-19 patients, and the risk of COVID-19 infection is higher in individuals with the CXCR2 rs2230054 C>T CT and (CT+CC) genotypes. In addition, the CC and CT genotypes have been demonstrated to increase the risk of mortality from COVID-19. However, our study did not show any differences in the allele frequency of the rs2230054 polymorphism between patients and controls. Current research on the relationship between the CXCR2 rs2230054 gene polymorphism and diseases is controversial. Our data are in agreement with the study reporting that the CXCR2 gene rs2230054 polymorphism is associated with peri-implantitis susceptibility in the Chinese Han population, and it was established that the CT genotype of rs2230054 serves as a risk factor for the occurrence of peri-implantitis [23]. However, Melanie *et al.* [19], who studied the association of IL-8, CXCR2, and TNF-α polymorphisms with COPD patients, observed that the CXCR2 +785 T allele may be important in protecting against pulmonary inflammation in these patients. Some reasons, such as immunogenetics and genetic structure differences between populations and different types of diseases, may explain the contradictory results of the studied IL-8 and CXCR2 gene polymorphisms.

### 4.4 Comparative Analysis of IL-10 (-1082 G>A), rs1800896 Gene Polymorphism

Regarding the IL-10 gene polymorphism, we found that the CA and CC genotypes as well as the C allele of the IL-10 (-1082 G>A), rs1800896 polymorphism are associated with high risk of infection: OR 2.0596 (1.1587 - 3.6609), OR 3.5(1.4505 - 8.445), and OR 2.0108 (1.3442 – 3.0081) respectively. Paradoxically, we found that the CA and CC genotypes of the IL-10 (-592) rs1800872 C>A polymorphism confer significant protection against mortality from COVID-19. This could be supported by the recent study conducted by Rizvi *et al.* [28], who found that the frequency of CA and AA genotypes and the A allele was higher among severe patients. The patients with CA, AA, and A alleles showed a higher risk of severity due to COVID-19 infection. However, their results failed to show any significant association. On the other hand, the frequency of occurrence of the CC genotype was found to be significantly lower in the severe group compared to the mild group, suggesting its protective role in decreasing the risk of COVID-19 severity. To the best of our knowledge, no research has been done to assess the relationship between COVID-19 patients' IL-8 rs4073 T>A polymorphism and CXCR2 rs2230054 C>T polymorphism. Additionally, there is little evidence in the literature to link the IL-10(-592) rs1800896 C>A polymorphism to Covid-19 severity and mortality.

In COVID-19 individuals who have a high risk of sickness and mortality, our research is the first of its type to examine several polymorphisms. Thus, we attempted to identify four relevant polymorphisms and their function in the risk of COVID-19 infection and survival in hospitalized COVID-19 patients. The SNPs considered had previously been shown to involve important chemokine pathways linked with COVID-19 pathophysiology, although the exact impact of polymorphisms in susceptibility and outcome in COVID-19 was unknown. As a result, our investigation provided commendable data to illustrate the impact of these polymorphisms in COVID-19 at the community level. However, we were able to generate results for IL-8 rs4073 T>A and for IL-10(-592) rs1800872 C>A polymorphisms in only 111 subjects out of the original 115 subjects involved. IL-10 -1082G/A appears to be associated with early or severe presentation of CAD. Further studies are warranted to confirm this association. In conventional meta-analyses, the results suggested that IL-10 -819C/T polymorphism was associated with decreased risk of CVD, especially CAD outcome, whereas IL-10 -592C/A and IL-10 -1082G/A polymorphisms might have no influence on the susceptibility of CVD [46-48].

Furthermore, conclusions from this study should only be extrapolated to the community with caution because it was conducted on hospital-based patients. This study had some weaknesses in addition to its strength in correlating these polymorphisms with the Covid-19 death rate in various SARS- CoV-2 variants. One of the major drawbacks of the study was the inability to compare the findings with healthy people without a history of Covid-19. The patients were from two different cities, and, as you know, in SA consanguinity plays a role in disease genetics. The sample size was modest. Moreover, we studied hospital mortality, and we did not know the outcome of survivors' re-admission.

## CONCLUSION

The findings of this study provide important insights into the Covid-19 disease and susceptibility risk. The polymorphisms in TNFα -308 G>A (rs1800629), IL-8, IL-10 (-1082 G>A) and CXCR2 +785 C>T are associated with the risk of susceptibility to Covid-19 and with mortality in Covid-19 patients. Further functional investigations may provide insight toward disease mechanisms for the development of more effective therapies. These results encourage further studies with larger sample sizes are necessary to confirm our findings.

## Figures and Tables

**Fig. (1):**
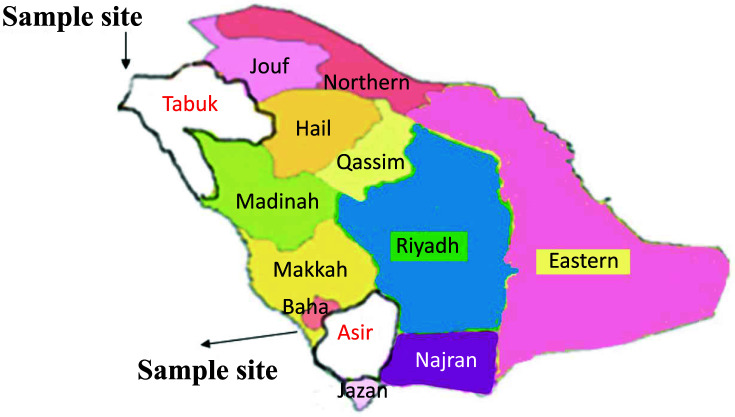
Map of Saudi Arabia by region. The two research regions of Tabuk and Asir are red labelled.

**Table 1:** Amplification refractory mutation system (ARMS) PCR primers for TNF-α, IL-8, IL-10, and CXCR2 genotyping.

**ARMS PCR Primers for TNFα -308 G>A (rs1800629) Genotyping**					
**Gene**	**Allele**	**Sequence**	**AT**	**PCR Product Size**	**References**
TNF*-*αF0	-	5`-ACCCAAACACAGGCCTCAGGACTCAACA-3`	62°C	323 bp	[32]
TNF*-*αR0	-	5`-TGGAGGCAATAGCTTTTGAGGGGCAGGA-3`	-	-	-
TNF*-*α FI	A allele	5`-AGTTGGGGACACGCAAGCATGAAGGATA-3`	-	154 bp	-
TNF*-*α RI	G allele	5`-TAGGACCCTGGAGGCTAGACCCCGTACC-3`	-	224 bp	-
**ARMS PCR Primers for IL-8 -251T>A (rs4073), Genotyping**					
IL8 -FO	-	5`- CATGATAGCATCTGTAATTAACTG-3`	58°C	349 bp	[32]
IL8 -RO	-	5`- CACAATTTGGTGAATTATCAAA-3`	-	-	-
IL8 -FI	A allele	5`- GTTATCTAGAAATAAAAAAGCATACAA-3`	-	169 bp	-
IL8 -RI	T allele	`5`-CTCATCTTTTCATTATGTCAGA-3`	-	228 bp	-
**ARMS PCR Primers for CXCR2 +785 C>T (rs2230054) Genotyping**					
CXCR2 FO	-	5`- CTGCCTGTCTTACTTTTCCGAAGGACCG-3`	63°C	451 bp	[19]
CXCR2 RO	-	5`- TCTTGAGGAGTCCATGGCGAAACTTCTG-3`	-	-	-
CXCR2 FI	C allele	5`- TCTTTGCTGTCGTCCTCATCTTCCTGATC-3`	-	226 bp	-
CXCR2 RI	T allele	5`- AGGACCAGGTTGTAGGGCAGCCAGAAA-3`	-	281 bp	-
**Allele Specific PCR Primers for IL-10 (-1082 G>A), rs1800896 Genotyping**					
IL-10 F1	A allele	5`-ACTACTAAGGCTTCTTTGGGA**A** -3	65°C	258 bp	[33]
IL-10 R	-	5`- CAGTGCCAACTGAGAATTGG -3`	-	-	-
IL-10 F2	G allele	5`- CTACTAAGGCTTCTTTGGGA**G** -3,	-	258 bp	-
IL-10 R	-	5`- CAGTGCCAACTGAGAATTGG -3`	-	-	-
Reference gene		5`- GCCTTCCCAACCATTCCCTTA -3`	-	429 bp	-
Reference gene		5`- TCACGGATTTCTGTTGTGTTTC-3`	-	-	-

**Table 2:** Clinical characteristics of the COVID-19 patients and healthy controls.

Clinical Feature	Value	**No. Patients (%)**	**Controls**
**-**	**-**	115	115
**Age (Years)**	>40	96(83.47%)	80(69.56%)
**-**	≤40	19(16.52%)	35(30.43%)
**Gender**	Male	69(60%)	90(78.26%)
**-**	Female	46(40%)	25(21.73%)
**Comorbidities**	-	-	-
CKD	-	11(9.40%)	-
T2DM	-	47(40.17%)	-
Hypertension	-	37(31.62%)	-
CAD	-	17(14.53%)	-
**Biochemical markers**	-	-	-
CRP	≥10 mg/l	94(81.73%)	-
ALT	≥36 U/l	73(63.47%)	-
AST	>40 U/l	78(67.82%)	-
Oxygen saturation	<80	63(54.78%)	-
Duration in hospital (days)	>30	56(48.69%)	-
**Treatment required**	-	-	-
Steroids	Steroids	99(86.08%)	-
Antiviral	Antiviral	114(99%)	-

**Table 3:** Comorbidities and COVID-19 outcome.

**Characteristics**	**Total (n=115)**	**Survivor** **(n=64)**	**Non-survivor** **(n=51)**	**Odd’s Ratio**	**Risk Ratio**	***p* value**
**Male**	69(60%)	33 (51.56%)	36 (70.58%)	1 (Ref.)	1 (Ref.)	0.02
**Female**	46(40%)	31 (48.44%)	15 (29.42%)	0.4435 (0.20-0.96)	0.62(0.389-1.002)	-
**Age >40**	96(83.47%)	54 (84.37%)	42 (82.35%)	1 (Ref.)	1 (Ref.)	**-**
**Age ≤40**	19(16.52%)	10(15.62%)	09 (17.30%)	0.8642 (0.322-2.318)	0.9236 (0.546-1.56)	0.482
**T2DM**	47 (40.17%)	08 (12.48%)	39 (76.47%)	22.75 (8.50- 60.834)	4.70 2.769-7.984)	< 0.0001
**Hypertension**	37 (31.62%)	06 (9.36%)	31 (72.54%)	14.98(5.450-41.187)	3.26(2.182-4.892)	< 0.0001
**CAD**	17 (14.53%)	00 (0)	17 (33.33%)	-	2.8(2.1967-3.782)	< 0.0001
**CKD**	11(9.4%)	2 (3.12%)	09 (17.64%)	6.642(1.36-32.298)	2.02(1.408-2.914)	< 0.009

**Table 4:** Genotype and allele frequency distribution for TNF-α rs1800629 G>A, IL-8 rs4073 T>A, IL-10 (-1082 G>A), rs1800896 and CXCR2 rs2230054 C>T in COVID-19 patients and control group.

**Subjects**	**N=**	**Genotypes**			**Df**	***X2***	**Allele**		***P value***
**Association of TNF-α rs1800629 G>A Genotypes and Alleles**					-	-	-	-	-
-		**GG**	**GA**	**AA**	-	-	**G**	**A**	-
**COVID-19 patients**	115	75(65.21%)	35(30.43%)	5 (4.34%)	2	7.31	0.80	0.20	0.025
**Healthy controls**	115	93(80.86%)	20(17.39%)	2 (1.73%)	-	-	0.90	0.10	-
**Association of IL-8 rs4073 T>A Genotypes and Alleles**					-	-	-	-	-
-		**TT**	**TA**	**AA**	-	-	**T**	**A**	-
**COVID-19 patients**	113	26(23.42%)	56(50.45%)	31(27.92%)	2	6.23	0.47	0.53	0.044
**Controls**	115	44(38.26%)	46(40%)	25(21.73%)	-	-	0.60	0.42	-
**Association of CXCR2 rs2230054 C>T Genotypes and Alleles**					-	-	-	-	-
-		**TT**	**CT**	**CC**	-	-	**T**	**C**	-
**COVID-19 patients**	115	30(26.08%)	77(66.95%)	8(6.95%)	2	6.54	0.59	0.41	0.038
**Controls**	115	48(41.73%)	59(51.30%)	8(6.95%)	-	-	0.77	0.33	-
**Association of IL-10 (-1082 G>A), rs1800896 Genotypes and Alleles**					-	-	-	-	-
-		**AA**	**CA**	**CC**	-	-	**A**	**C**	-
**COVID-19 patients**	111	40(30.03%)	51(45.94%)	20(18%)	2	10.91	0.59	0.41	0.004
**Controls**	111	63(56.75%)	39(35.13%)	9(8.10%)	-	-	0.75	0.25	-

**Table 5:** Logistic regression analysis of TNF-α rs1800629 G>A gene polymorphism using different inheritance models for prediction of susceptibility to COVID-19.

**Genotypes**	**COVID-19 Patients (N=115)**	**Control Group** **(N=115)**	**Odd Ratio** **OR (95% CI)**	**Risk Ratio** **RR (95% CI)**	***P* value**
**Codominant Inheritance Model**			-	-	-
TNF-α-GG	75	93	1(Ref.)	1(Ref.)	-
TNF-α-GA	35	20	2.17(1.158- 4.066)	1.42(1.097-1.851)	0.01
TNF-α-AA	5	2	3.1(0.58 – 16.43)	1.6 (0.9725-2.632)	0.157
**Dominant Inheritance Model**			-	-	-
TNF-α-GG	75	93	1(Ref.)	1(Ref.)	-
TNF-α (GA+AA)	40	22	2.25(1.234-4.119)	1.44 (1.125 - 1.855)	0.005
**Recessive Inheritance Model**			-	-	-
TNF-α -(GG+GA)	110	113	1(Ref.)	1(Ref.)	-
TNF-α-AA	5	2	2.56(0.48 to 13.51	1.44(0.8897-2.3467)	0.26
**Allele**	-	-	-	-	-
TNF-α-G	185	206	1(Ref.)	1(Ref.)	-
TNF-α-A	45	24	2.08(1.22-3.5603)	1.378(1.126–1.68)	0.006
**Over Dominant Inheritance Model**			-	-	-
TNF-α-(GG+AA)	80	95	1(Ref.)	1(Ref.)	-
TNF-α (GA)	35	20	2.07(1.112 to 3.81)	1.39(1.076 to 1.79)	0.021

**Table 6:** Logistic regression analysis of IL-8 rs4073 T>A gene polymorphism using different inheritance models for prediction of susceptibility to COVID-19.

**Genotypes**	**Control Group (N=115)**	**COVID-19 ** **Patients (N=111)**	**Odd Ratio** **OR (95% CI)**	**Risk Ratio** **RR (95% CI)**	***P* value**
Codominant Inheritance Model					
IL-8- TT	44	26	1(Ref.)	1(Ref.)	-
IL-8- TA	46	54	1.98(1.0641 to 3.7088)	1.36(1.0344 to 1.8052)	0.031
IL-8- AA	25	31	2.09(1.0255 - 4.2939)	1.40(0.9994 to 1.9837)	0.042
**Dominant Inheritance Model**					
IL-8- TT	44	26	1(Ref.)	1(Ref.)	-
IL-8- (TA+AA)	71	85	2.02(1.1363 to 3.6122)	1.38 (1.0769 to 1.7713)	0.016
**Recessive Inheritance Model**					
IL-8 -(TT+TA)	90	80	1(Ref.)	1(Ref.)	**-**
IL-8- AA	25	31	1.39 (0.7604 to 2.5594)	1.18 (0.8575 to 1.6401)	0.28
**Allele**					
IL-8 -T	134	106	1(Ref.)	1(Ref.)	-
IL-8 -A	96	116	1.52(1.0535 to 2.2149)	1.23(1.0238 to 1.4849)	0.025
**Over Dominant Inheritance Model**					
IL-8 -(TT+AA)	69	57	1(Ref.)	1(Ref.)	-
IL-8- (TA)	46	54	1.42(0.8390 to 2.4070)	1.19 (0.9132 to 1.5519)	0.191

**Table 7:** Logistic regression analysis of CXCR2 rs2230054 C>T gene polymorphism using different inheritance models for prediction of susceptibility to COVID-19.

**Genotypes**			**Control Group** **(N=115)**		**COVID-19 Patients (N=115)**	**Odd Ratio** **OR (95% CI)**			**Risk Ratio** **RR (95% CI)**					***P* Value**
**Codominant Inheritance model**														
CXCR2-TT			48		30	1(Ref.)			1(Ref.)					-
CXCR2-CT			59		77	2.0(1.1842 to 3.681)			1.41(1.094 – 1.836)					0.011
CXCR2-CC			8		8	1.60(0.543 to 4.711)			1.23(0.7314 to 2.0711)					0.394
**Dominant Inheritance Model**														
CXCR2-TT			48		30	1(Ref.)			1(Ref.)					-
CXCR2 (CT+CC)			67		85	2.0(1.162 to 3.54)			1.39(1.0865- 1.793)					0.012
**Recessive Inheritance Model**														
CXCR2 -(TT+CT)			107		107	1(Ref.)			1(Ref.)					-
CXCR2-CC			8		8	1.0(0.3620 to 2.76)			1.10(0.6017 to 1.6619)					1.00
**Allele**			-		-	-			-					-
CXCR2-T			155		137	1(Ref.)			1(Ref.)					-
CXCR2-C			8		8	1.13(0.4136 to 3.094)			1.06(0.6403 - 1.7509)					0.810
**Over Dominant Inheritance Model**														
CXCR2-(CC+TT)			56		38	1(Ref.)			1(Ref.)					-
CXCR2 (CT)			59		77	1.92(1.128 to 3.27)			1.37(1.0650 to 1.7706)					0.016
**Association of IL-10 (-1082 G>A), rs1800896 Genotypes and Alleles**														
-		**AA**		**CA**			**CC**	-		-	**A**	**C**	-	
**COVID-19 patients**	111	40(30.03%)		51(45.94%)			20(18%)	2		10.91	0.59	0.41	0.004	
**Controls**	111	63(56.75%)		39(35.13%)			9(8.10%)	-		-	0.75	0.25	-	

**Table 8:** Logistic regression analysis of IL-10 (-1082 G>A), rs1800896 gene polymorphism using different inheritance models for prediction of susceptibility to COVID-19.

**Genotypes**	**Control Group** **(N=111)**	**COVID-19 Patients** **(N=111)**	**Odd Ratio** **OR (95% CI)**	**Risk Ratio** **RR (95% CI)**	***P* Value**
**Codominant Inheritance Model**					
IL-10- AA	40	63	1(Ref.)	1(Ref.)	-
IL-10- GA	51	39	2.0596(1.1587 - 3.6609)	1.4592(1.0785 - 1.9742)	0.009
IL-10- GG	20	9	3.5(1.4505 - 8.445)	1.7759(1.2589 – 2.5051)	0.003
**Dominant Inheritance Model**					
IL-10- AA	40	63	(Ref.)	1(Ref.)	-
IL-10- (GA+GG)	71	48	2.3297(1.3582 - 3.9961)	1.5363(1.1367 – 2.0406)	0.001
**Recessive Inheritance Model**					
IL-10 -(GA+AA)	91	102	1(Ref.)	1(Ref.)	-
IL-10- GG	20	9	2.4908(1.0797 - 5.746)	1.4627 (1.0986 – 1.9474)	0.022
**Allele**					
IL-10 –A	131	165	1(Ref.)	1(Ref.)	-
IL-10 -G	91	57	2.0108 (1.3442 – 3.0081)	1.3893 (1.1598 - 1.6643)	< 0.001
**Over Dominant Inheritance Model**					
IL-10 -(AA+GG)	60	72	1(Ref.)	1(Ref.)	-
IL-10- (GA)	51	39	1.5692 (0.9148 - 2.6918)	1.2467 (0.9613 - 1.6167)	0.066

**Table 9:** General characteristics of COVID-19 patients between survivors and non survivors.

**Characteristics**	**Total (n=115)**	**Survivor** **(n=64)**	**Non-survivor** **(n=51)**	**Odd’s Ratio**	**Risk Ratio**	***p* value**
**CKD**	11(9.40%)	02 (3.12%)	09 (17.64%)	6.642(1.36-32.298)	2.02(1.408-2.914)	< 0.009
**T2DM**	47 (40.17%)	08 (12.48%)	39 (76.47%)	22.75 (8.50- 60.834)	4.70 2.769-7.984)	< 0.0001
**Hypertension**	37 (31.62%)	06 (9.36%)	31 (72.54%)	14.98(5.450-41.187)	3.26(2.182-4.892)	< 0.0001
**CAD**	17 (14.53%)	00 (0)	17 (33.33%)	-	2.8(2.1967-3.782)	< 0.0001
**Age >40**	96(83.47%)	54 (84.37%)	42 (82.35%)	1 (Ref.)	1 (Ref.)	**-**
**Age ≤40**	19(16.52%)	10(15.62%)	09 (17.30%)	0.8642 (0.322-2.318)	0.9236 (0.546-1.56)	0.482
**Male**	69(60%)	33 (51.56%)	36 (70.58%)	1 (Ref.)	1 (Ref.)	0.02
**Female**	46(40%)	31 (48.44%)	15 (29.42%)	0.4435 (0.20-0.96)	0.62(0.389-1.002)	-
**Oxygen saturation <80**	12(18.75%)	-	51 (100%)	-	-	< 0.0001
**Duration in hospital (days) >30**	13(20.31%)	-	43 (84.31%)	21.0 (7.99-55.61)	5.6(2.926-10.958)	< 0.0001
**CRP≥10 mg/l**	104 (97.44%)	43 (67.18%)	51 (100%)	-	-	< 0.0001
**ALT≥36 U/l**	45 (38.57%)	26 (40.62%)	47 (92.15%)	17.17 (5.513-53.49)	6.7(2.620-17.439)	< 0.0001
**AST>40 U/l**	48 (41.3%)	22 (34.37%)	46 (90.19%)	17.56(6.101-50.559)	6.3(2.7322-14.799)	< 0.0001
**Steroids**	77 (65.81%)	48 (75%)	51 (100%)	-	-	< 0.0001
**Antiviral**	79 (67.52%)	64 (100%)	51(100%)	-	-	< 0.00001

**Table 10:** Haplotype association with response (n=212, crude analysis).

-	**SNP1**	**SNP2**	**SNP3**	**Freq**	**OR (95% CI)**	***P*-value**
1	T	T	G	0.4864	1.00	-
2	A	C	G	0.3102	0.03 (0.01 - 0.14)	< 0.0001
3	A	T	G	0.0667	0.56 (0.21 - 1.50)	0.25
4	T	T	A	0.0398	0.00 (-Inf - Inf)	1
5	A	T	A	0.0368	5.38 (1.21 - 23.93)	0.028
6	A	C	A	0.0297	0.22 (0.06 - 0.78)	0.02
7	T	C	G	0.0188	0.00 (-Inf - Inf)	1
8	T	C	A	0.0116	0.00 (-Inf - Inf)	1
Global haplotype association *p*-value: < 0.0001						

## Data Availability

The authors confirm that the data supporting the findings of this research are available within the article.

## LIST OF ABBREVIATIONS

ANOVA: Analysis of Variance
CAD: Coronary Artery Disease
CIs: Confidence Intervals
CKD: Chronic Kidney Disease
COVID-19: Coronavirus Disease-2019
CXCR-2: CXC Chemokine Receptor-2
HWD: Hardy-Weinberg Disequilibrium
HWE: Hardy-Weinberg Equilibrium
IL-10: Interleukin 10
IL-8: Interleukin 8
OD: Optical Density
ORs: Odd Ratios
PASC: Post-acute Sequelae of COVID-19
RRs: Risk Ratios
SARS-COV-2: Severe Acute Respiratory Syndrome Coronavirus -2
SNPs: Single-nucleotide Polymorphisms
T2DM: Type 2 Diabetes Mellitus
TNF-α: Tumor Necrosis Factor Alpha

## STANDARDS OF REPORTING

STROBE guidelines were followed.
